# Supplementary material for: Metagenome-assembled genomes from a population-based cohort uncover novel gut species and within-species diversity, revealing prevalent disease associations
Source: mSystems. 2026 Mar 16;11(4):e00114-26. doi: 10.1128/msystems.00114-26 (PMC13098258; doi:10.1128/msystems.00114-26)
Supplement: Supplemental Figures — Fig. S1 to S6. [file msystems.00114-26-s0001.docx]

**Supplementary Figures**


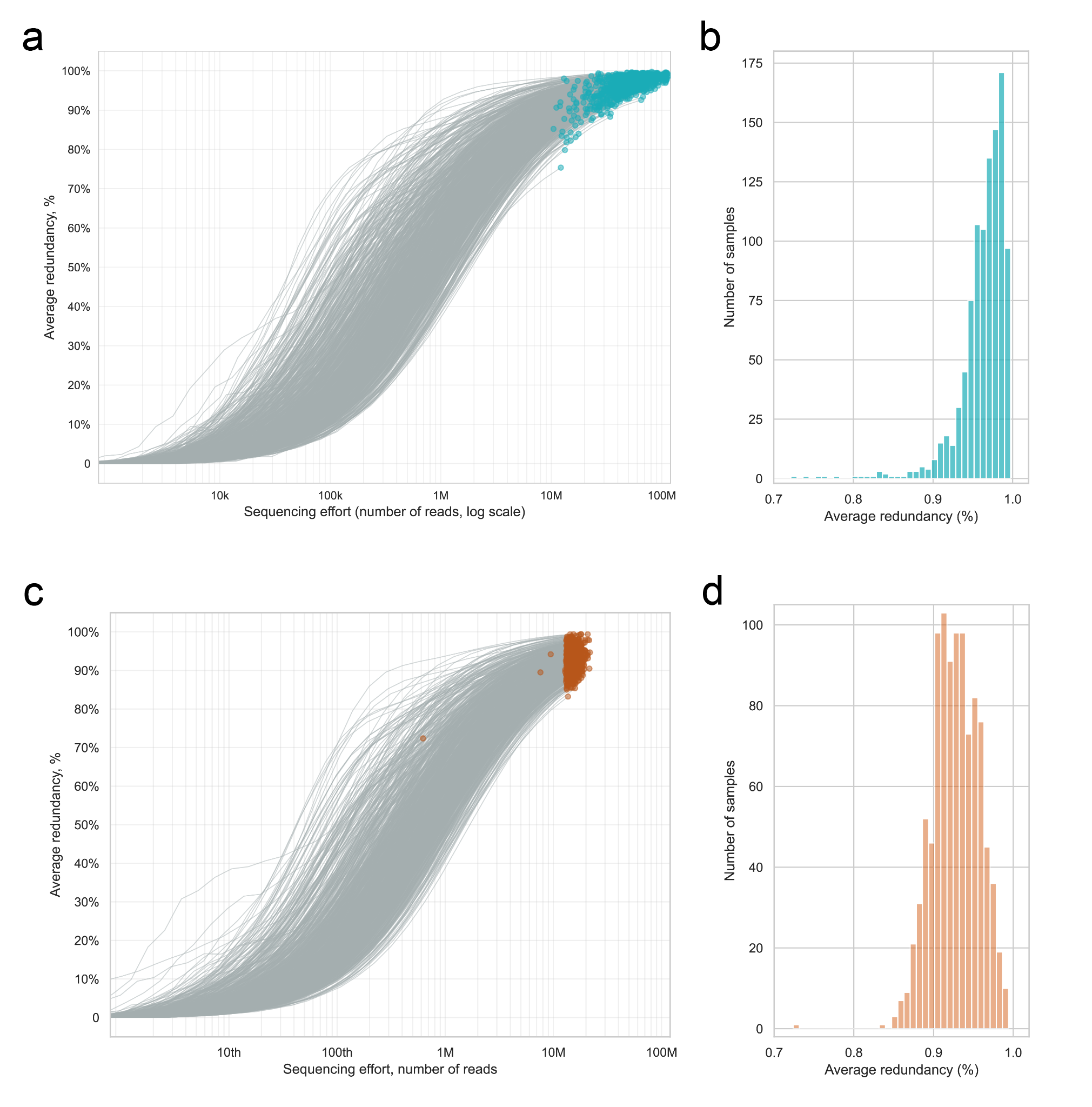


**Figure S1.** Nonpareil-based estimates of metagenomic sequencing coverage**. a.** Redundancy curves for EstMB-deep samples. **b.** Distribution of average redundancy across EstMB-deep samples. **c.** Redundancy curves for EstMB samples. **d.** Distribution of average redundancy across EstMB samples.

**
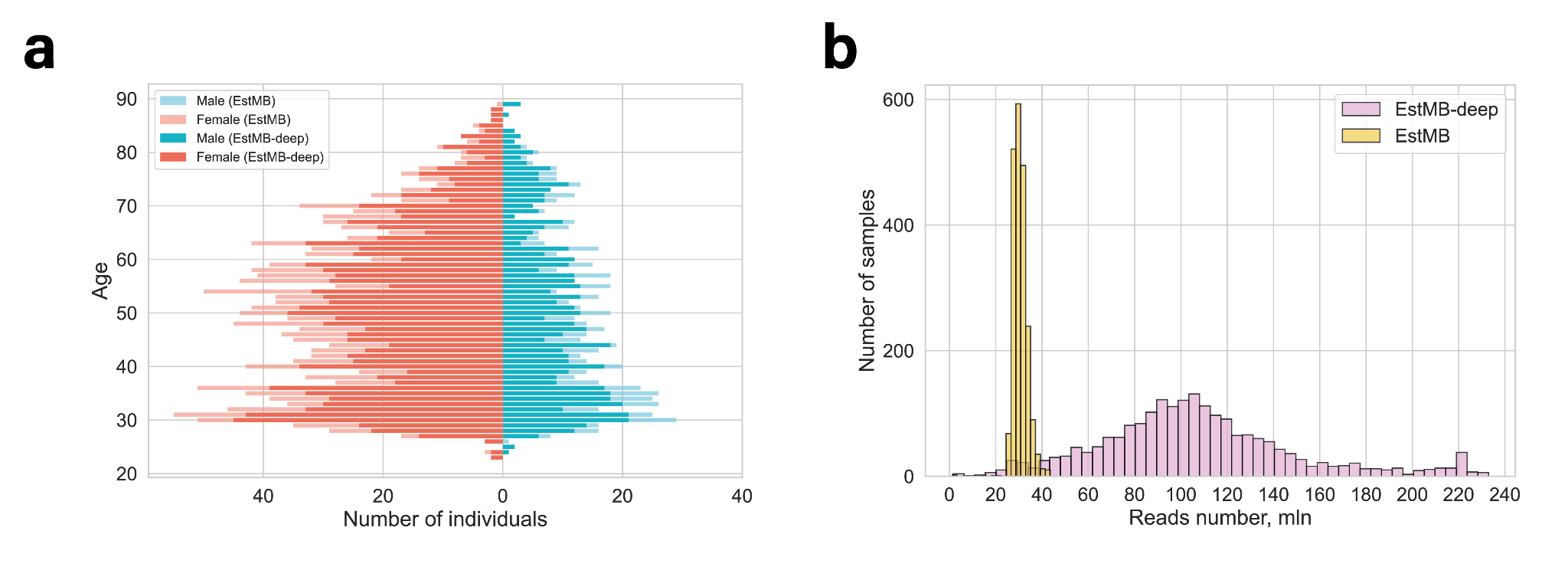
**

**Figure S2.** Cohort demographics and sequencing depth in EstMB and EstMB-deep **a.** Distribution of age and gender across the Estonian Microbiome cohort (EstMB) and Estonian Microbiome deep sequencing cohort (EstMB-deep). **b.** Distribution of the number of reads across different genders of EstMB and EstMB-deep cohorts.


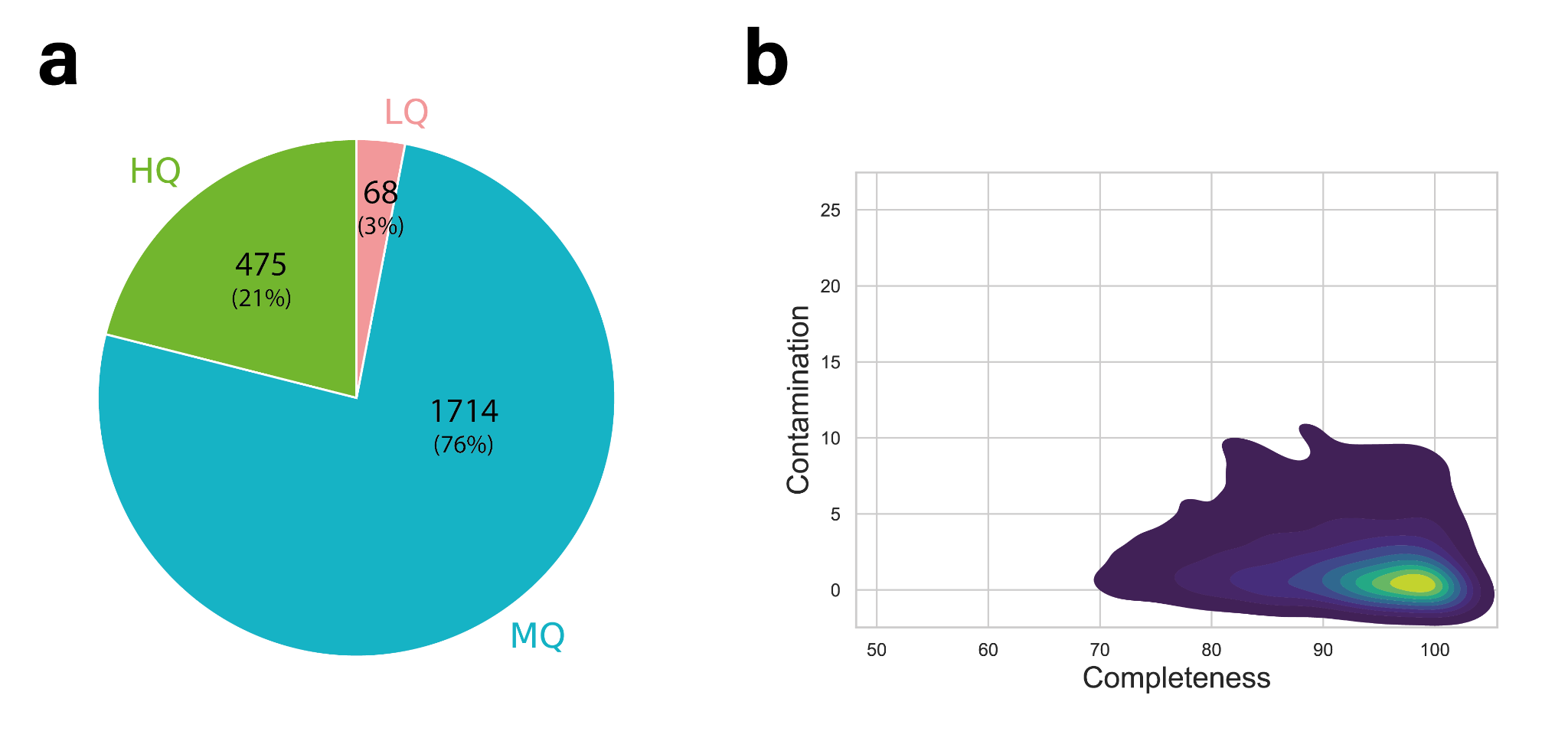


**Figure S3.** Quality estimation of species representative MAGs. **a.** Quality distribution of ESTrep MAGs (HQ, high quality; MQ, medium quality; and LQ, low quality). **b.** Completeness and contamination density profiles of ESTrep MAGs.


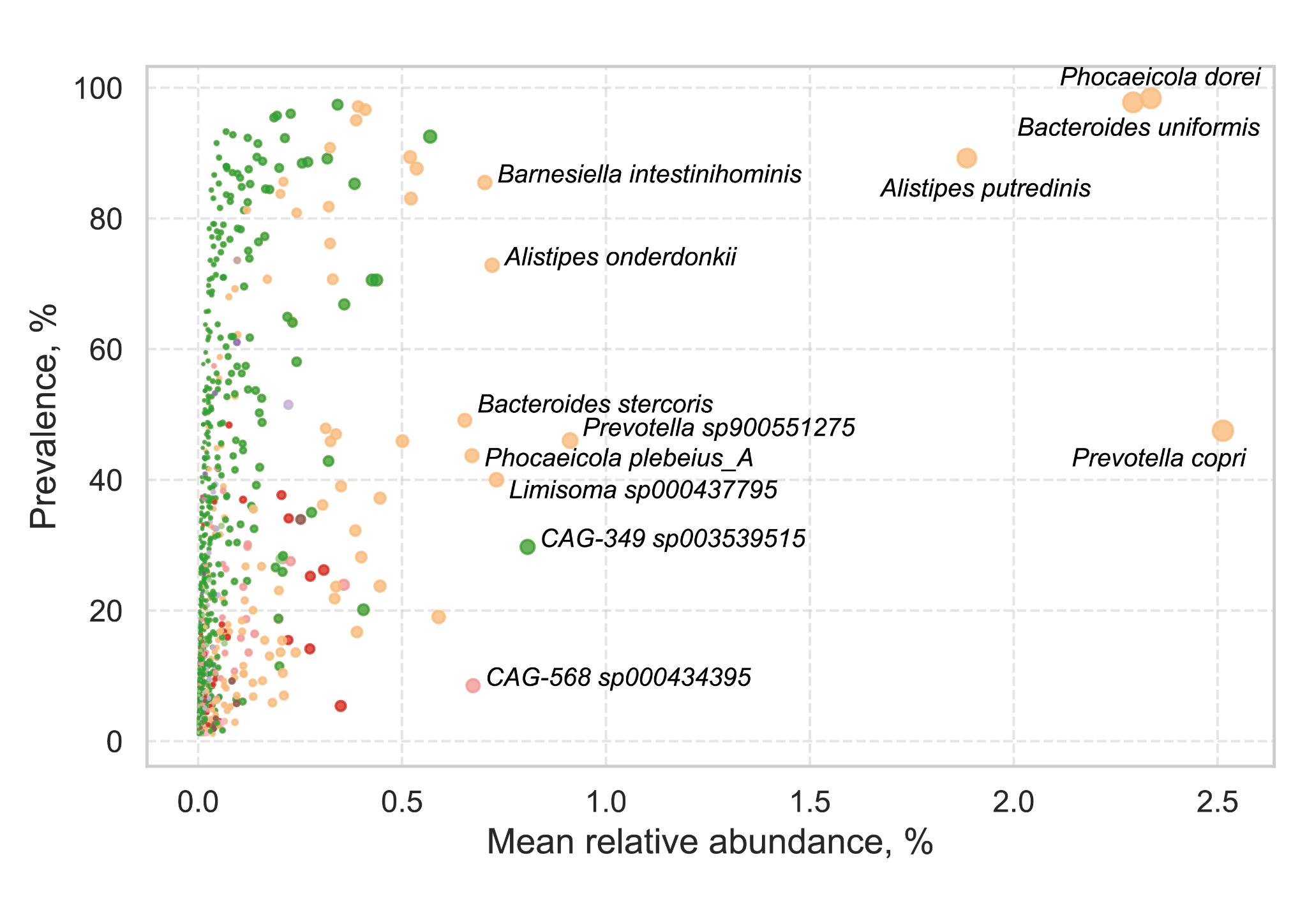


**Figure S4.** The ESTrep species prevalence and mean relative abundance. Species with the highest mean relative abundance are labelled.


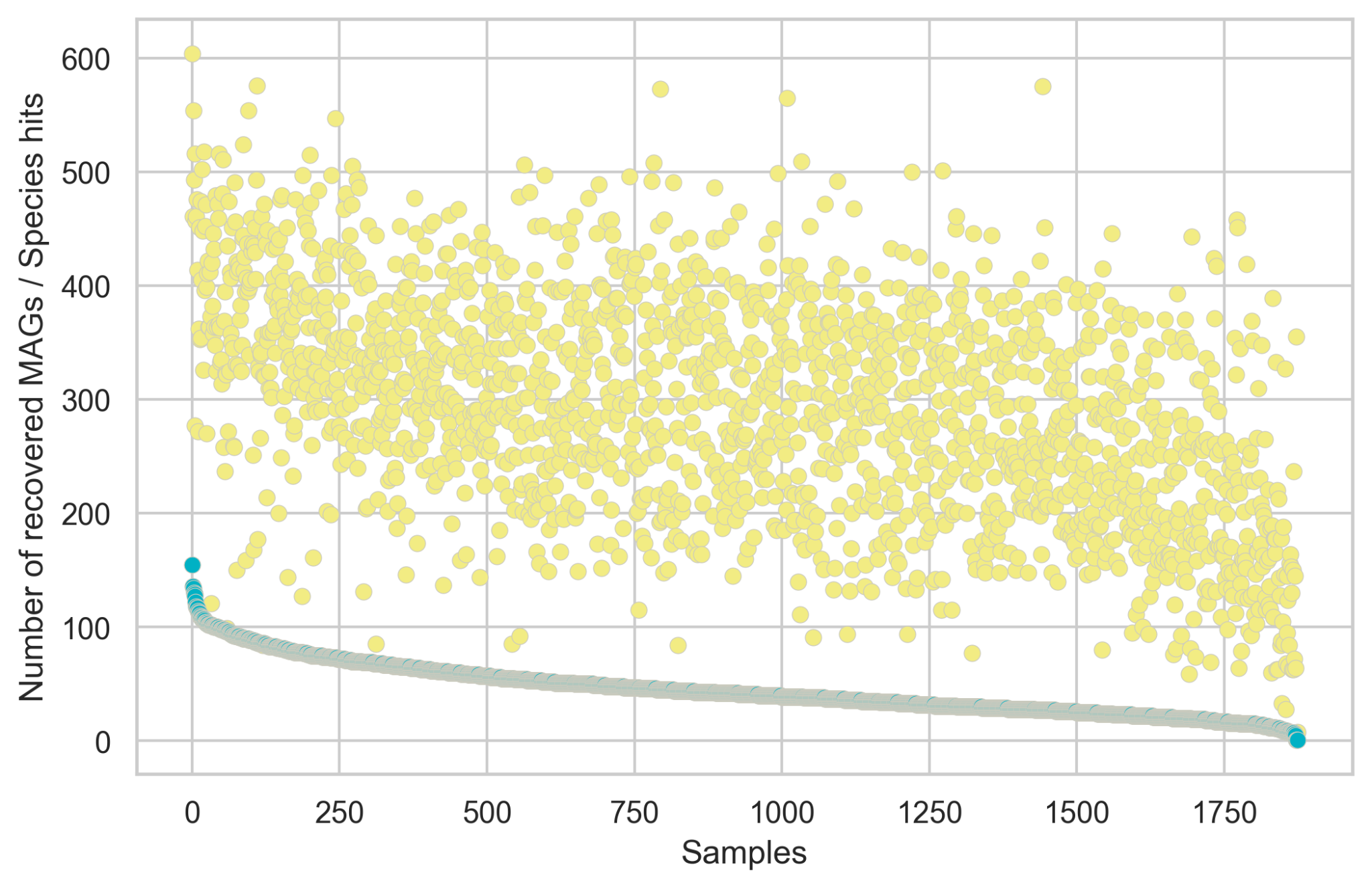


**Figure S5**. Number of detected species by read mapping (yellow) and number of recovered MAGs (blue/grey) per sample.


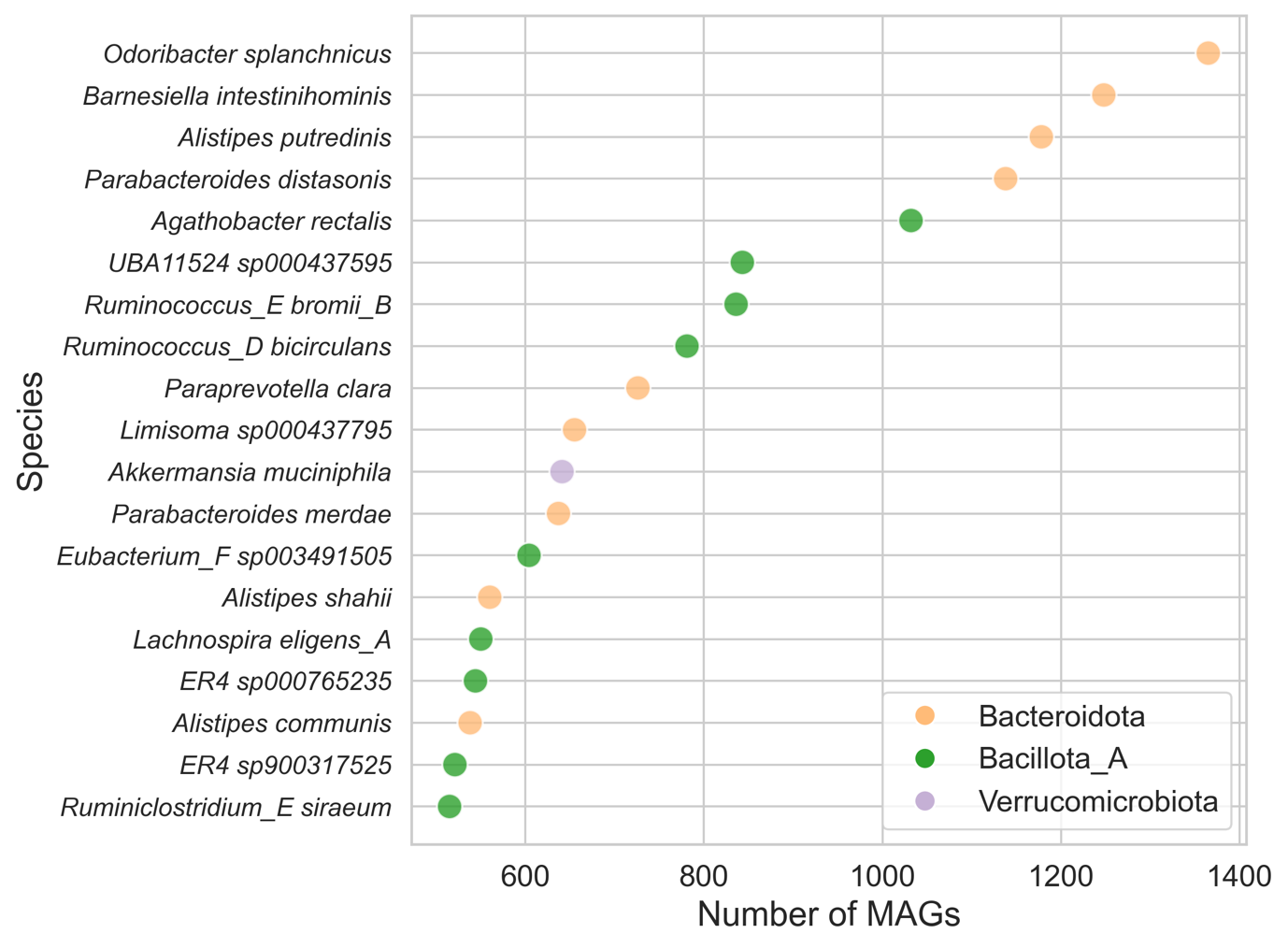


**Figure S6.** Species with the highest number of recovered MAGs
